# Weight for Length/Height Percentiles in Infants and Young Children in Kayseri/Turkey

**DOI:** 10.4274/Jcrpe.1139

**Published:** 2013-12-12

**Authors:** M. Mümtaz Mazıcıoğlu, Türev Demirtaş, Betül Çicek, Ahmet Öztürk, Selim Kurtoğlu, Hasan Basri Üstünbaş

**Affiliations:** 1 Erciyes University, Faculty of Medicine, Department of Pediatric Endocrinology, Kayseri, Turkey; 2 TOKİ Family Health Center, Kayseri, Turkey; 3 Erciyes University, Faculty of Health Sciences, Kayseri, Turkey

**Keywords:** Length, height, weight, children

## Abstract

**Objective:** To produce weight for length/height (WLH) percentiles to be used for the screening of growth and assessment of failure to thrive in infancy and early childhood.

**Methods:** The data (2009-2010) of the Anthropometry of Turkish Children aged 0-6 years (ATCA-06) study were used. A cross-sectional study was designed to calculate the WLH references. Reference weight values for each 5-cm LH intervals were determined using the LMS Chart Maker Pro version 2.3 software program (The Institute of Child Health, London).

**Results:** A total of 3123 children (1573 female, 1550 male) aged 0-6 years were included in the calculation of the 3rd, 5th, 10th, 25th, 50th, 75th, 85th, 90th, 95th, and 97th WLH percentiles. The difference between the 3rd and the 97th percentiles for males was 2.02 cm to 12.64 cm in the 50-54.99 cm and 125-130 cm LH ranges. In the girls, the differences between the 3rd-97th percentiles ranged from 2.02 cm to 12.64 cm in the 50-54.99 cm and 125-130 cm LH groups. The maximum difference between the 3rd and 97th percentiles was about half the variation of mean WLH throughout the first six years of life. The most rapid change in WLH was observed in the 0-2-year period. Turkish references for WLH were not different from the World Health Organization standards.

**Conclusions:** This is the first study in Turkey presenting WLH references in 0-6 year old children. We suggest that the use of WLH in the first two years of life may be more useful than age-adjusted references in assessment of nutritional status and diagnosis of failure to thrive.

**Conflict of interest:**None declared.

## INTRODUCTION

Frequent screening of weight changes in early childhood is primarily a reliable indicator of normal nutrition, undernutrition, or obesity. Body mass index (BMI), which is derived from weight and length/height (LH), is the primary universal parameter used to define obesity by showing body frame size, lean and fat tissue content in a single index. However, length (in those under age 2 years) or standing height (in those older than age 2 years) also need to be taken into account in the evaluation of body weight. The use of weight for LH (WLH) percentiles in the diagnosis of failure to thrive (FTT) has already been reported (1). In the assessment of nutritional status, it should be remembered that LH loss is significantly less than weight loss (6% vs. 31%, respectively) particularly in the early stages of undernutrition (2,3). WLH provides the weight spectrum for a specific length segment (4,5).Therefore, the use of BMI as the key index for nutritional evaluation may not be as satisfactory in children under two years of age as it is in older children and adults (6). The age-adjusted references of several anthropometric measurements are currently used as either follow-up or screening criteria to monitor growth in children (7).

In the last four decades, age-adjusted references of several anthropometric measurements of Turkish children and adolescents have been published. These references have been updated in recent years, and also, data on new anthropometric measurements have been added (8,9,10).

This study aims to contribute to the estimation of obesity and FTT in early childhood by providing reference data for WLH in Turkish children. 

## METHODS

The data of the Anthropometry of Turkish Children Aged 0-6 years (ATCA-06) study in 2009-2010 were used to calculate the WLH references. Cross-sectional sampling was performed from the health records of the Family Health Centers (FHC) located in the city center and suburbs of Kayseri, a city with more than 1 200 000 inhabitants. The data sampling units were 21 FHC located in the city center and suburbs of Kayseri. Stratification according to the socioeconomic status of parents and sampling proportional with the population in each socioeconomic level (low, medium, and high) was performed as recommended by the local educational authority.

Infants with conditions like prematurity and low birth weight or multiple births, and those who had any known chronic or serious illness or malnutrition which may interfere with growth were excluded. The study protocol was approved by the Ethics Committee of Erciyes University, and individual consent was obtained from the parents.

All measurements were performed by two well-trained health technicians. Inter-observer correlation coefficients were calculated as ≥0.98. The weight and height measurements were taken in duplicate, and the average of the two measurements was used. An electronic digital scale (Seca, 354; accurate to 10 g) was used to weigh children aged 0-2 years without clothing and in a dry diaper. In children over 2 years, weight measurement was conducted with a standard beam balance scale (Tefal Ultraslim, France; accurate to 100 g) with children wearing only underwear. Length was measured in children aged less than 2 years by two examiners (one to position the child) with the child supine on a measuring board. Height was measured with a portable stadiometer which was calibrated daily in children who were over two years old. Chronological age was calculated as the completed three months intervals according to their exact date of birth. Construction of the percentiles of the 0-6 year olds for LH in 5 cm intervals was done with the least mean squares (LMS) Chart Maker Pro version 2.3 software program (The Institute of Child Health, London), which fits smooth centiles to reference data using the LMS method (11). 

## RESULTS

A total of 3024 children (1535 female, 1489 male) aged between 0 and 6 years were included in the study. The age distribution of infants and children from birth to six years was 671 (22.2%), 505 (16.7%), 422 (14.0%), 385 (12.7%), 378 (12.5%), 412 (13.6%), and 251 (8.3%), respectively.

The minimum increment in the mean LH from birth to age 6 years was 52.9 cm in boys and 54.0 cm in girls. The percentile values for LH-adjusted weight are given in Tables 1 and 2. The difference between the 3rd-97th percentile values for boys ranged from 2.02 to 11.76 kg in the 50-54.99 cm and 125-130 cm LH groups, respectively. In the girls, the differences between the 3rd-97th percentiles ranged from 2.02 cm to 12.64 cm in the 50-54.99 cm and 125-130 cm LH groups.

The LH-adjusted weight increment in the 50th percentile was 18.59 kg for boys and 20.11 kg for girls. The increment in the 50th percentile for each 5-cm interval in the WLH between the shortest and tallest children ranged from 0.97-1.87 kg for boys and 1.11-1.55 kg for girls. The increment for the 3rd and 97th percentiles in boys was 15.65 cm and 25.38 cm, respectively, and in girls, it was 17.14 cm and 26.95 cm, respectively. The range in LH in each age and gender group was about 23-35 cm in our sample. In Tables 1 and 2, the 3rd, 5th, 10th, 25th, 50th, 75th, 85th, 90th, 95th, and 97th WLH percentiles and growth curves for 0-6-year-old male and female children are shown. The calculated age-adjusted length/height cut-offs for weight in both genders are shown in Table 3. We found that the correlation between LH and weight is 94.4, but the age-adjusted correlation was 72.9.

## DISCUSSION

LH, weight, and head circumference are the three principal anthropometric measurements used to assess growth, particularly in early childhood. Although age and gender are the primary criteria used to assess the anthropometric measurements of particular children, evaluation of weight with reference to LH (WLH), instead of age (weight for age) is proposed to make a significant contribution in diagnosing FTT in infancy and early childhood (2,3,4,5).

In spite of the fact that LH is a denominator in the calculation of BMI, age-adjusted BMI calculation may not be specific for the discrimination of lean or body fat mass (2,3,4,5). Stature-dependent BMI in different periods has the potential for variation by the relative leg length and sitting height to different directions (4,12). Therefore, screening growth and nutrition together with WLH may provide additional reliable information in early childhood.

There are a number of studies which suggest that adolescent and adult obesity are related to LH in childhood (13).

To our knowledge, this study provides the first LH references in a group of 0-6-year-old Turkish children. Our findings indicate that in early childhood, there is no significant difference between the genders in the final change of WLH, but there is a great difference between the upper and lower limits of weight for a particular range of LH, which is 23.5 cm in girls and 24 cm in males. This difference may be used as a gross and easily recalled parameter to assess growth monitoring. The maximum difference between the 3rd-97th percentiles is about half the variation of the mean WLH in the first six years of life.

We used the minimum and maximum LH as the lower and higher limits for our references, 50-130 cm for both genders. The relatively small sample size in our study prevented us from calculating LH percentiles in a much narrower range (e.g. 1 cm intervals). Cross-sectional study design may also be considered as a limitation since serial measurements over a period of time would allow more precise results in the assessment of growth (14). However, since the difference in weight for each 5-cm interval is about 1.5 kg, we consider that a 5-cm range would not result in a significant deviation.

**Figure 1:**
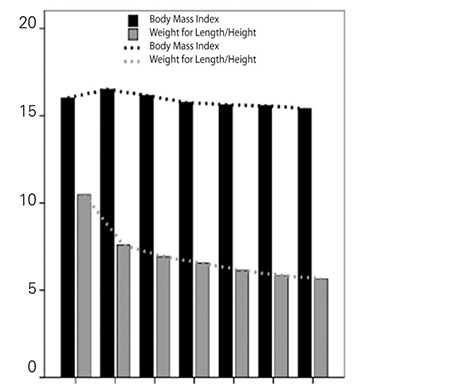
Comparison of variation in body mass index and weight for length/height ratios in preschool children

**Figure 2:**
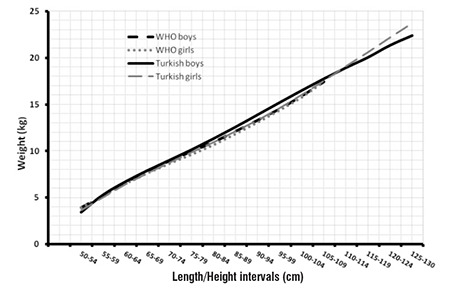
The comparison of 50th percentiles of weight for length/height between World Health Organization standards and Turkish group

When changes in BMI and WLH values in infants and children aged less than 6 years are compared, it is noted that no significant change occurs in BMI values with age, other than a small increment in the second year of life (Figure 1). On the other hand, a rapid decrease in WLH ratio is noted after the first year of life, a finding which is probably related to rapid growth in length in this age group. These findings indicate that WLH could be considered a reliable method to monitor growth in infants and young children and also suggest that LH-adjusted weight may be a more reliable index than age-adjusted weight when comparing national data with international standards. As shown in Figure 2, the WLH values for Turkish children were found to be comparable to World Health Organization references (14,15).

**Figure 3:**
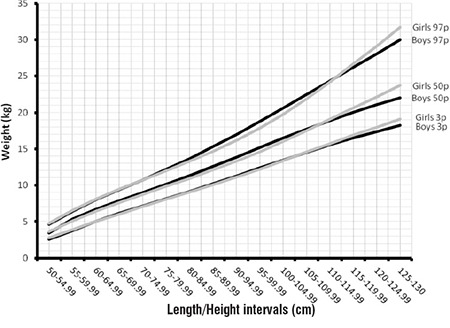
The comparison of 3rd, 50th and 97th percentiles of weight for length/height between the genders in infants and children

In Figure 3, we compared the 3rd, 50th and 97th percentiles of WLH in girls and boys. In the 50th percentile and the 3rd and 97th percentiles, there were no significant differences between the two genders for WLH in LH groups under 110 cm. In the 110-130 cm range, the height-adjusted weight difference between girls and boys gradually increased to 1.0-1.5 cm. This difference may be explained by the observation that obesity rebound is higher in girls than in boys.

In conclusion, we believe that our findings may be used as the first WLH references in our population. Since WLH is relatively high in the first 24 months of life, interpretation of weight should be made according to LH primarily in this age group. Since the 5th percentile value for LH-adjusted weight is considered a prerequisite in the early diagnosis of FTT, these reference values could be helpful in the diagnosis of FTT in Turkish children. 

## ACKNOWLEDGEMENTS

We would like to thank the local health authority for their assistance in data collection for this article.

## Figures and Tables

**Table 1:**
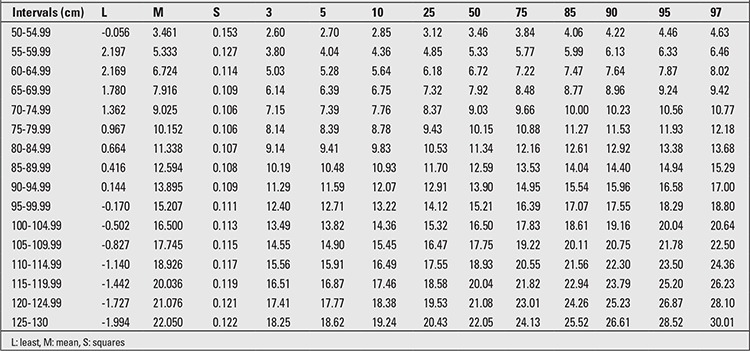
The 3rd, 5th, 10th, 25th, 50th, 75th, 85th, 90th, 95th, 97th length/height-adjusted weight percentiles of 0-6-year-old boys

**Table 2:**
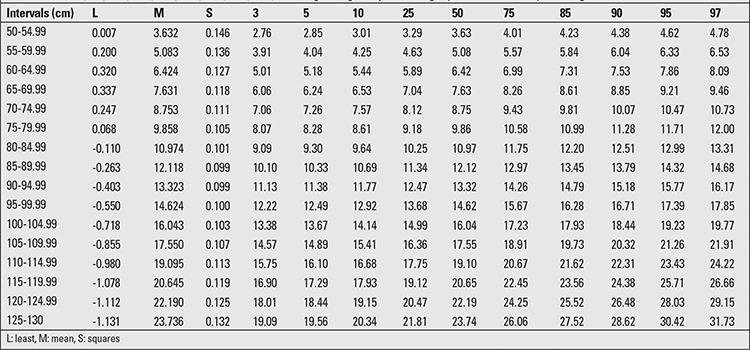
The 3rd, 5th, 10th, 25th, 50th, 75th, 85th, 90th, 95th, 97th length/height-adjusted weight percentiles of 0-6-year-old girls

**Table 3:**
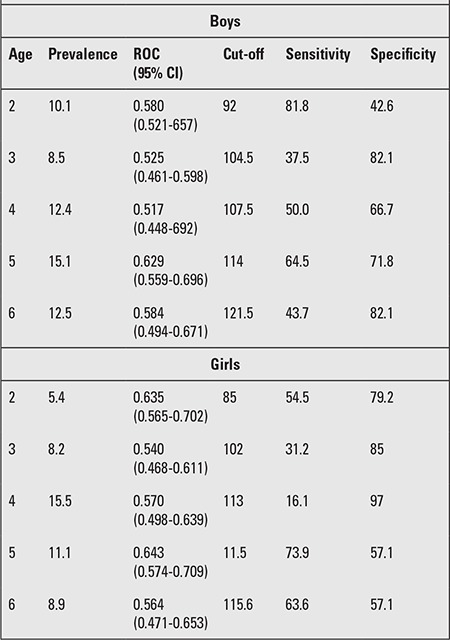
Age-adjusted length/height cut-offs for weight in both genders

## References

[1] Olsen EM (2006). Failure to thrive: still a problem of definition. Clin Pediatr (Phila).

[2] Cole SZ, Lanham JS (2011). Failure to thrive: an update. Am Fam Physician.

[3] Bertagnon JR, Dall Colletto GM (2003). Weight-for-length relationship at birth to predict neonatal diseases. Sao Paulo Med J.

[4] Garn SM, Leonard WR, Hawthorne VM (1986). Three limitations of the body mass index. Am J Clin Nutr.

[5] Mandel D, Zimlichman E, Mimouni FB, Grotto I, Kreiss Y (2004). Height-related changes in body mass index: a reappraisal. J Am Coll Nutr.

[6] Barlow SE, Expert Committee (2007). Expert committee recommendations regarding the prevention, assessment, and treatment of child and adolescent overweight and obesity. Pediatrics.

[7] Taveras EM, Rifas-Shiman SL, Sherry B, Oken E, Haines J, Kleinman K, Rich-Edwards JW, Gillman MW (2011). Crossing growth percentiles in infancy and risk of obesity in childhood. Arch Pediatr Adolesc Med.

[8] Gokcay G, Furman A, Neyzi O (2008). Updated growth curves for Turkish children aged 15 days to 60 months. Child Care Health Dev.

[9] Altunay C, Kondolot M, Poyrazoglu S, Mazicioglu MM, Kurtoglu S (2011). Weight and height percentiles for 0-84-month-old children in Kayseri-A central Anatolian city in Turkey. J Clin Res Pediatr Endocrinol.

[10] Hatipoglu N, Kurtoglu S, Ozturk A, Mazicioglu MM (2009). The weight and height percentiles in 6-18 year old children in Kayseri and comparison with Istanbul data. J Clin Res Pediatr Endocrinol.

[11] Cole TJ, Green PJ (1992). Smoothing reference centile curves: the LMS method and penalized likelihood. Stat Med.

[12] Pawson IG, Huicho L, Muro M, Pacheco A (2001). Growth of children in two economically diverse Peruvian high-altitude communities. Am J Hum Biol.

[13] Freedman DS, Khan LK, Mei Z, Dietz WH, Srinivasan SR, Berenson GS (2002). Relation of childhood height to obesity among adults: the Bogalusa heart study. Pediatrics.

[14] Winch EA (2002). Obtaining accurate growth measurements in children. J Spec Pediatr Nurs.

[15] Kondolot M, Balci E, Ozturk A, Mazicioglu MM, Hatipoglu N, Kurtoglu S, Ustunbas HB (2011). Body mass index percentiles for Turkish children aged 0-84 months. Ann Hum Biol.

